# Intermolecular Interactions of Linear Alkane (*N* ≤ 18) Dimers: A High Accuracy Theoretical Study

**DOI:** 10.1021/acsomega.5c11053

**Published:** 2026-05-04

**Authors:** Chenhui Wang, WanYing Huang, Liang Pu, Zhong Zhang, Robert Bruce King

**Affiliations:** † College of Chemistry & Pharmacy, 12469 Northwest A&F University, Yangling, Shaanxi 712100, P. R. China; ‡ Department of Chemistry and Center for Computational Chemistry, 1355 University of Georgia, Athens, Georgia 30602, United States

## Abstract

This work systematically investigates the intermolecular interactions in *n*-alkane (C_*n*_H_2*n*+2_, *n* = 1–18) dimers through high-accuracy DLPNO–CCSD­(T1)/CBS­(aug2/3)//M05-2X-D3/6-31G** calculations. The relative errors of interaction energies are below 5% with respect to CCSD­(T)/CBS­(aug-cc-2/3)//M05-2X-D3/6-31G** benchmarks. The BSSE-corrected results are reported from −2.2 kJ/mol (*n* = 1) to −62.6 kJ/mol (*n* = 18). The interaction energy (Δ*E*) increases linearly with chain length (*n*), as quantified by the equation Δ*E*(*n*) = −3.6*n* + 2.2 kJ/mol, providing a more accurate quantitative description compared to the previous MP2/CBS research. Moreover, thermodynamic analysis reveals that spontaneous dimerization starts from *n* ≥ 8 at 100 K. These findings provide a high-accuracy benchmark for noncovalent interactions in *n*-alkane dimers, deepening the understanding of van der Waals interaction strength, and providing valuable insights for molecular design.

## Graphical abstract

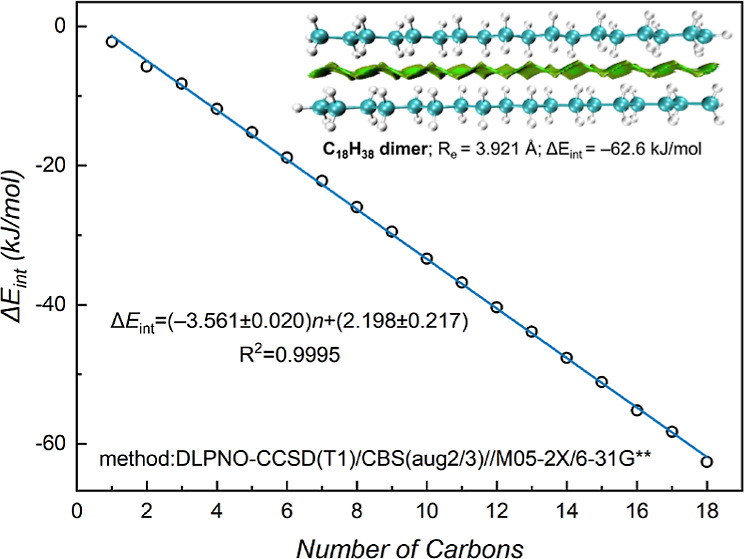

## 1 Introduction

Chemical reactions are mainly dominated by covalent thermodynamics. Thus, the well-known white phosphorus breaks into two P_2_ units at elevated temperatures,^1^ because the 2P_2_ → P_4_ process is a thermodynamically spontaneous reaction. In contrast, the ubiquitous noncovalent intermolecular interactions have been regarded as less important for chemical reactions. Recently, the importance of noncovalent intermolecular interactions has been clearly recognized as a frontier area of chemical research. The London dispersion force (LDF),^2,3^ as a part of van der Waals forces, arises from the attraction of instantaneous molecular multipoles originating from electron dynamic correlation. It is considered the weakest of intermolecular forces, typically being less than 1 kcal/mol between light atoms. However, the LDF increases with atomic number as well as with molecular size, leading to a significant overall stabilization energy of tens of kcal/mol between large molecules because of its cumulative effect. Therefore, LDFs can become relevant in asymmetric catalysis,^4–6^ the stabilization of novel inorganic and organometallic molecules,^1,2,7,8^ supramolecular chemistry,^9–11^ pharmaceutical design,^12^ and understanding crystalline materials.^7^

The accurate prediction of LDF is a notorious theoretical challenge, since LDF originates from long-range dynamic electron correction effects. While computational quantum methods are very powerful tools for quantitative analysis, density functional theory (DFT) methods are notoriously deficient in describing such interactions.^13^ These inaccuracies are inherent to the functional and cannot be resolved simply by adjusting the basis set size.^14^ Although empirical corrections like D3 ^15^ and D4 ^16^ have improved the accuracy of the interaction structures and energies to a certain extent, this represents only a small step toward the accuracy required for quantitative studies.^17^ On the other hand, the CCSD­(T)^18^ method is considered the gold standard method for calculating noncovalent interaction energies.^19^ However, its formidable computational cost renders it prohibitive for larger systems.^20^ The MP2/CBS approach offers an alternative approach. However, it exhibits significant deviations from the CCSD­(T)/CBS benchmark, with the relative error reaching up to 11% even for a system as small as the ethane dimer.^21^ Recently, the near linear scaling DLPNO–CCSD­(T) method,^22,23^ was developed by Neese’s group to contain 99.9% E_C_ of canonical CCSD­(T) for accurate and effective calculations. To achieve higher chemical accuracy, particularly in systems dominated by dispersion interactions, the DLPNO–CCSD­(T1) method^24^ was subsequently developed, incorporating an improved perturbative triples correction.

The LDF in *n*-alkanes (C_*n*_H_2*n*+2_) is a typical example for several reasons. First, each *n*-alkane has too many conformations owing to free rotation around −CH_2_–CH_2_–, even leading to the folded C_18_H_38_ conformation at 100 K.^25–27^ Therefore, the different conformation–conformation interactions lead to so-called “fluxional conformations”. Additionally, the variable structure of *n*-alkane aggregations leads to so-called “fluxional structures”. For example, the methane dimer has six structures with the D_3d_ global minimum.^28–30^ This LDF complicated system is critically important for advancing our understanding of intermolecular interactions, yet it also presents a significant challenge for quantitative theoretical description.

To address this challenge, this work employs a robust computational strategy, aiming to establish a reliable benchmark data set of interaction energies for *n*-alkane (C_*n*_H_2*n*+2_, *n* = 1–18) dimers with near-CCSD­(T)/CBS accuracy. We seek to elucidate how the interaction energy changes with chain length and to explore the thermodynamic conditions for spontaneous dimerization. These findings are anticipated not only to quantify the strength and physical origin of van der Waals interactions in these fundamental systems but also to establish a quantitative foundation for leveraging dispersion interactions as a predictive tool in the rational design of novel molecules and reactions in fields such as drug development and materials science.

## 2 Computational Methods

For the precise calculation of intermolecular noncovalent interaction energies with greater than chemical accuracy, two fundamental challenges must be overcome. One challenge is the reliability of geometry optimization for reducing energetic error from geometric deviation. The other challenge is that a nearly perfect computational method must be chosen in order to achieve very tiny energy errors in single-point energy calculations.

In 1990, Szczesniak et al.^28^ discovered that the most stable structure of methane dimer has D_3d_ point group symmetry, confirmed by Tsuzuki and co-workers.^31,32^ In subsequent studies, Tsuzuki et al.^33,34^ further pointed out that the most stable structures of propane and *n*-pentane dimers possess C_2h_ point group symmetry, while the most stable structures of *n*-butane and *n*-hexane dimers have D_2_ point group symmetry. Therefore, the structural optimization of *n*-alkane dimers follows the above-mentioned point group rule.

Grimme and Steinmetz^35^ showed that the B2PLYP-D3/QZVP method further enhances the accuracy of molecular structure calculations. However, the B2PLYP-D3/QZVP method is extremely expensive for larger *n*-alkane dimers. Because of this high cost, we required a different method for this work.

In order to calculate accurately the interaction energies of *n*-alkane dimers, high-level electron correlation methods are required. The CCSD­(T) method is considered the gold standard for calculating noncovalent interaction energies. However, its high O­(N^7^) scaling and large basis set requirement render it computationally prohibitive for the systems in this work.^36^ Therefore, to achieve comparable accuracy to the canonical CCSD­(T) method with high computational efficiency, the DLPNO–CCSD­(T1) and DLPNO–CCSD­(T) approaches were evaluated. The aug-cc-pVXZ (X = D, T)^37,38^ basis set was selected because its diffuse functions are essential for capturing noncovalent interactions.^39^ The EP2 ^40–43^ method was then applied for energy extrapolation to the complete basis set (CBS) limit.

The interaction energy (Δ*E*_int_) of the *n*-alkane dimers was decomposed using the local energy decomposition (LED)^44^ scheme. Within the LED framework, Δ*E*_int_ is hierarchically decomposed: first into the Hartree–Fock (Δ*E*_HF_) and correlation (Δ*E*_C_) components. The latter is then partitioned into the perturbative triple (Δ*E*_(T)_) and CCSD (Δ*E*_CCSD_) contributions. Finally, the CCSD term is divided into dispersion (Δ*E*_disp_) and nondispersion (Δ*E*_nondisp_) components. This decomposition is given by

$${\Delta E}_{\mathrm{int}}={\Delta E}_{HF}+{\Delta E}_{C}$$

$${\Delta E}_{C}={\Delta E}_{\left(T\right)}+{\Delta E}_{CCSD}$$

$${\Delta E}_{CCSD}={\Delta E}_{disp}+{\Delta E}_{non-disp}$$

Thermodynamic corrections were calculated at the B3LYP-D3­(BJ)/6-31G* ^15,45–49^ level. The zero-point energy was scaled by a factor of 0.977.^50^ Then the vibrational entropy was treated with a frequency threshold of 100 cm^–1^ according to the protocol established in ref 51. The rationale for this computational strategy is provided in the Supporting Information.

All quantum chemical calculations were performed using the following software: Gaussian 09 ^52^ for Density Functional Theory (DFT), ORCA^53^ for DLPNO–CCSD­(T), and Shermo^54^ for thermodynamic corrections. The DFT calculations employed the D3 dispersion correction with the Becke-Johnson damping scheme.^55^ The DLPNO–CCSD­(T1) and DLPNO–CCSD­(T) calculations^56^ employed the default RI approximation. Specifically, the “TightPNO” setting was used for DLPNO–CCSD­(T1), while the “NormalPNO” setting was applied along with a “TcutPairs” value of 10^–5^ for DLPNO–CCSD­(T). The basis set superposition error (BSSE) was corrected using the counterpoise method of Boys and Bernardi.^53,57^ Based on the electron density obtained from DFT calculations, noncovalent interaction (NCI) isosurfaces were plotted at a reduced density gradient (RDG) value of 0.65 au using Multiwfn (v3.8)^58^ and visualized with VMD (v1.9.3).^59^

## 3 Results and Discussion

### 3.1 Geometry Optimization

The methane dimer was employed as a model system for benchmark geometry optimization methods for the larger *n*-alkane dimers, as detailed in the Supporting Information. The M05-2X-D3/6-31G**^60^ method was identified as optimal against the gold-standard CCSD­(T)/aug-cc-pVTZ reference, exhibiting tiny structural deviation while maintaining high computational efficiency. It was therefore selected for this study.

Interaction energy curves were computed to assess further the accuracy and transferability of the M05-2X-D3/6-31G** method. A potential energy scan for the methane dimer at this level was performed: one monomer was fixed at its optimized geometry, and the interaction energy was calculated as the second monomer approached from 5.0 Å in steps of 0.1 Å. For benchmarking, this scan was also performed using the high-accuracy DLPNO–CCSD­(T1)/CBS­(aug2/3) method (Figure 1a). The same protocol was then applied to the *n*-butane dimer to demonstrate the applicability of the M05-2X-D3/6-31G** method to larger *n*-alkane dimers (Figure 1b).

**Figure 1:**
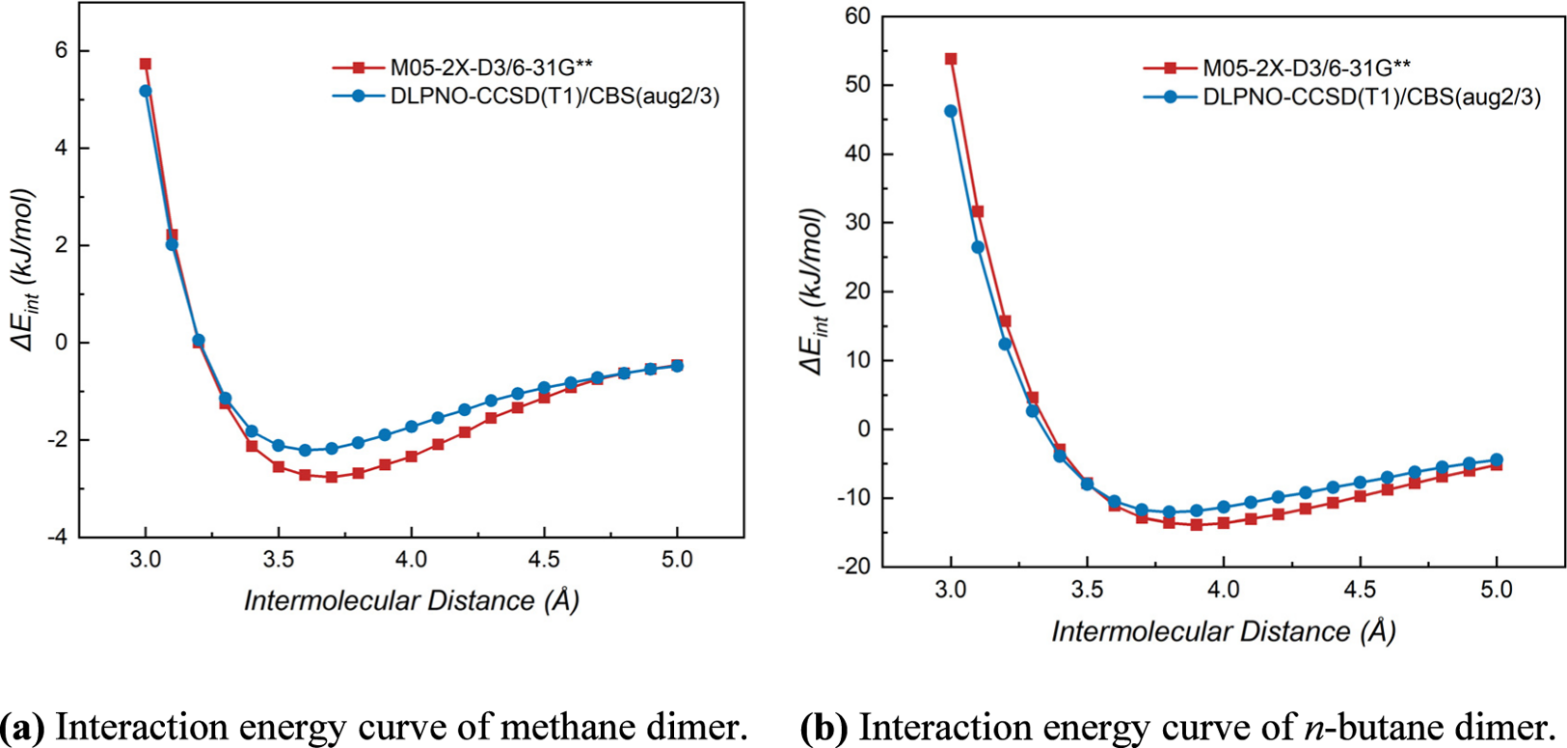
Interaction energy (Δ*E*_int_) curves of different *n*-alkane dimers.

The equilibrium distance (*R*_e_) values predicted by the M05-2X-D3/6-31G** method for the methane and *n*-butane dimers are in close agreement with the DLPNO–CCSD­(T1)/CBS­(aug2/3) benchmarks (Table S1 of the Supporting Information and Figure 1): 3.658 Å vs 3.65 ± 0.05 Å for methane and 3.899 Å vs 3.85 ± 0.05 Å for *n*-butane. This indicates that both methods locate the minimum-energy structures at nearly identical positions on the potential energy surface. Additionally, the *R*_e_ of methane dimer is optimized by CCSD/aug-cc-pVTZ to be 3.695 Å, very close to the M05-2X-D3/6-31G** value of 3.658 Å with a small deviation of 0.037 Å. Therefore, the M05-2X-D3/6-31G** method provides reliable structures for *n*-alkane dimers. However, the M05-2X-D3/6-31G** method significantly overestimates the interaction energies (Δ*E*_int_) compared to the DLPNO–CCSD­(T1)/CBS­(aug2/3) benchmarks: approximately −2.8 kJ/mol vs −2.2 kJ/mol (27% higher) for methane and approximately −13.9 kJ/mol vs −12.0 kJ/mol (16% higher) for *n*-butane. This indicates that the method is excellent for geometry optimization but has fundamental limitations in computing interaction energies. Consequently, using the DLPNO–CCSD­(T1)/CBS­(aug2/3)//M05-2X-D3/6-31G** method is essential for obtaining accurate interaction energies for *n*-alkane dimers.

### 3.2 Presentation of Structures

The geometry optimization of *n*-alkane (C_*n*_H_2*n*+2_, *n* = 1–18) dimers was carried out using the M05-2X-D3/6-31G** method without any imaginary frequencies, confirming that all optimized structures are true minima. The NCI plots visualizing the noncovalent interactions for the representative optimized structures (*n* = 1–6) are shown in Figure 2, with the corresponding plots for the larger dimers (*n* = 7–18) provided in Figure S1 of the Supporting Information.

**Figure 2:**
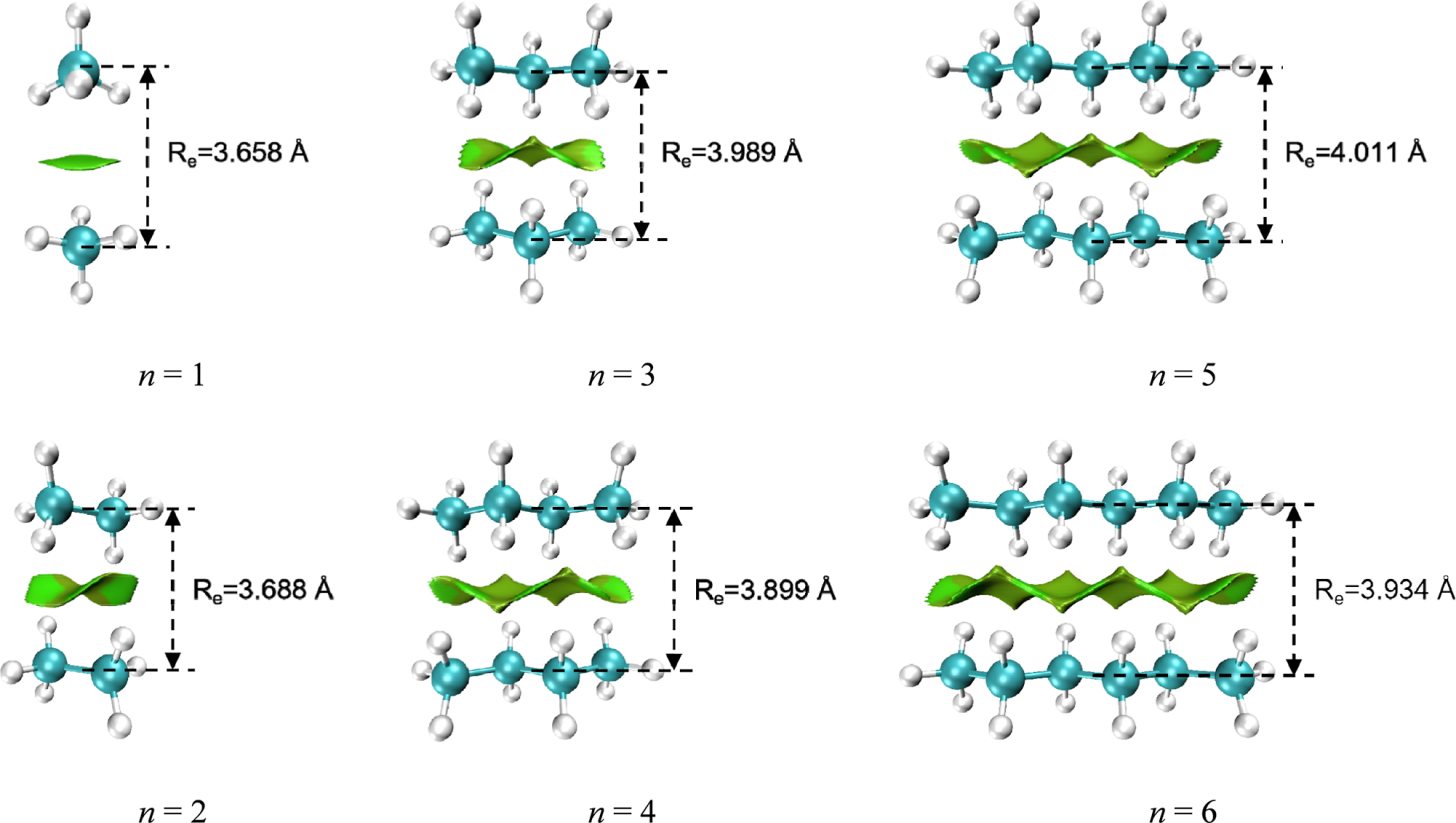
NCI plots of representative *n*-alkane (C_*n*_H_2*n*+2_, *n* = 1–6) dimers. The equilibrium distances were given in angstrom (Å). The color scale is based on sign­(λ_2_)­ρ, ranging from blue (strong attraction) through green (weak dispersion) to red (strong repulsion).

The noncovalent interaction region between the *n*-alkane (C_*n*_H_2*n*+2_, *n* = 1–18) dimers expands systematically with increasing chain length (Figures 2 and S1 in the Supporting Information). Turning to the geometry, the equilibrium distances (*R*_e_) of the methane and ethane dimers are distinctly shorter, at 3.658 Å and 3.688 Å, respectively. In contrast, the *R*_e_ values for larger dimers (*n* ≥ 3) fall within a range of 3.899–4.056 Å. A clear odd–even difference is observed among these larger dimers: the maximum *R*_e_ value (3.949 Å) for even *n*-alkane dimers is less than the minimum *R*_e_ value (3.989 Å) for odd *n*-alkane dimers. We attribute the difference to the configuration at the chain midpoint where *R*_e_ is measured. In even *n*-alkane dimers, this midpoint lies at a C–C bond. In odd *n*-alkane dimers, however, this site can be approximated as two opposing methylene groups, generating greater steric hindrance and thus a longer *R*_e_ compared to the even *n*-alkane dimers.

### 3.3 Interaction Energy Calculation and Benchmarking

Based on the optimized structures, the interaction energies of *n*-alkane (C_*n*_H_2*n*+2_, *n* = 1–4) dimers were calculated at the DLPNO–CCSD­(T1)/CBS­(aug2/3) and DLPNO–CCSD­(T)/CBS­(aug2/3) levels (Table 1). For benchmarking, the CCSD­(T)/CBS­(aug2/3) values based on the optimized structures and the gold-standard CCSD­(T)/CBS values from ref 21 are also listed.

**Table 1:** Interaction Energies (in kJ/mol) of *n*-Alkane (C_*n*_H_2*n*+2_, *n* = 1–4) Dimers: DLPNO–CCSD­(T1)/CBS Versus DLPNO–CCSD­(T)/CBS Versus CCSD­(T)/CBS

method	*n* = 1	*n* = 2	*n* = 3	*n* = 4
DLPNO–CCSD(T1)^a^	–2.2	–5.8	–8.2	–11.9
DLPNO–CCSD(T)^a^	–2.1	–5.5	–7.9	–11.3
CCSD(T)^a^	–2.3	–6.0	–8.6	–12.3
CCSD(T)^b^	–2.1	–5.1	–7.8	–11.5

a Basis set is aug-cc-pVXZ (X = D, T).

b Values are from ref 21.

Compared to the CCSD­(T)/CBS//M05-2X-D3/6-31G** benchmark, the DLPNO–CCSD­(T1)/CBS and DLPNO–CCSD­(T)/CBS methods respectively exhibit absolute deviations of less than 0.4 and 1.0 kJ/mol and relative errors below 5% and 9% for all dimers (Table 1). These results clearly indicate that DLPNO–CCSD­(T1)/CBS provides superior accuracy, making it the best choice for obtaining precise interaction energies. However, this gain in accuracy incurs a significant computational cost. For the methane-to-butane dimer series, the DLPNO–CCSD­(T1)/CBS completes calculations 1.4 to 2.5 times slower than DLPNO–CCSD­(T)/CBS (Table S2 in the Supporting Information), a performance gap widening with system size. Thus, DLPNO–CCSD­(T)/CBS offers a favorable efficiency-accuracy trade-off, especially for larger systems. Additionally, the Δ*E*_int_ values of CCSD­(T)/CBS//M05-2X-D3/6-31G** in Table 1 are generally larger than the corresponding values of the CCSD­(T)/CBS benchmark from ref 21. For instance, the Δ*E*_int_ of butane dimer was predicted by CCSD­(T)/CBS//M05-2X-D3/6-31G** and CCSD­(T)/CBS//MP2/6-311G** to be −12.3 kJ/mol and −11.5 kJ/mol, respectively. This difference may originate from a structural difference between M05-2X-D3/6-31G** and MP2/6-311G**, or originate from the different extrapolation strategies. However, the *R*_e_ of butane dimer was reported to be 3.899 Å (M05-2X-D3/6-31G**) and 3.903 Å (MP2/6-311G**). Such a tiny structural difference leads to the same Δ*E*_int_ of −11.9 kJ/mol at the DLPNO–CCSD­(T1)/CBS level. Therefore, we suspected that Helgaker’s^41^ extrapolation method applied in ref 21 and Neese’s improved EP2 method^40^ used in this paper leads to the slight differences of Δ*E*_int_ values.

Based on these findings, the DLPNO–CCSD­(T1)/CBS­(aug2/3)//M05-2X-D3/6-31G** method was adopted for this study to prioritize precision. For future investigations targeting more complex or numerous systems, the DLPNO–CCSD­(T)/CBS­(aug2/3)//M05-2X-D3/6-31G** method presents an efficient alternative.

### 3.4 Interaction Energies of *n*-Alkane Dimers

Following the benchmark studies on smaller dimers, the interaction energies for the complete series (C_*n*_H_2*n*+2_, *n* = 1–18) were computed at the DLPNO–CCSD­(T1)/CBS­(aug2/3) level (Table 2).

**Table 2:** Interaction Energies (Δ*E*_int_, kJ/mol) of *n*-Alkane (C_*n*_H_2*n*+2_, *n* = 1–18) Dimers at the DLPNO–CCSD­(T1)/CBS­(aug2/3)//M05-2X-D3/6-31G** Level

Dimer	*n* = 1	*n* = 2	*n* = 3	*n* = 4	*n* = 5	*n* = 6	*n* = 7	*n* = 8	*n* = 9
Δ*E*_int_	–2.2	–5.8	–8.2	–11.9	–15.2	–18.9	–22.2	–26.0	–29.5

Dimer	*n* = 10	*n* = 11	*n* = 12	*n* = 13	*n* = 14	*n* = 15	*n* = 16	*n* = 17	*n* = 18
Δ*E*_int_	–33.4	–36.8	–40.4	–43.9	–47.6	–51.1	–55.2	–58.3	–62.6

The |Δ*E*_int_| increases by an average of +3.6 kJ/mol per additional methylene group (Table 2). Notably, this increase exhibits a subtle odd–even oscillation: the gain is larger for the extension from an odd to an even *n*-alkane (avg. + 3.8 kJ/mol) than from an even to an odd *n*-alkane (avg. + 3.3 kJ/mol). Overall, the |Δ*E*_int_| of *n*-alkane dimers increases monotonically with carbon chain length, reaching a maximum of 62.6 kJ/mol for the C_18_H_38_ dimer. Significant errors are found to occur if the CBS extrapolation method is not used and BSSE is not corrected, as indicated by the 18.2% overestimation of the |Δ*E*_int_| for the C_18_H_38_ dimer calculated using the aug-cc-pVTZ basis (Table S3 in the Supporting Information). This value (62.6 kJ/mol) is substantially greater than the typical energy of an intermolecular hydrogen bond (e.g., 20.8 kJ/mol in water dimers^61^). This suggests that the role of van der Waals interactions in molecular chemistry may have been severely underestimated. Indeed, London dispersion forces can shift our view of steric effects from solely repulsive to newly recognized attractive.^2^ An example is the “hexaphenylethane riddle”: hexaphenylethane is too sterically crowded to be synthetically accessible, but all-*meta*-*tert*-butylhexaphenylethane is stable. This is attributed to the overall dispersion attraction (257.8 kJ/mol) provided by the *tert*-butyl groups, overcoming the repulsion and making the net steric effect attractively dominated.

A clear nonadditive trend is observed: the |Δ*E*_int_| of an even *n*-alkane dimer exceeds twice that of the corresponding *n*-alkane dimer with half the chain length. For example, |Δ*E*_int_| for the C_4_H_10_ dimer is 11.9 kJ/mol, exceeding twice the value (5.8 kJ/mol) for the C_2_H_6_ dimer (i.e., |Δ*E*_int_(C_4_H_10_)| > 2 × |Δ*E*_int_(C_2_H_6_)|). Elsewhere, the synergistic cooperativity^62^ effect becomes more obvious as the size of dimer enlarging, i. e. |Δ*E*_int_(C_4_H_10_)| – 2 × |Δ*E*_int_(C_2_H_6_)| = 0.3 kJ/mol, |Δ*E*_int_(C_14_H_30_)| – 2 × |Δ*E*_int_(C_7_H_16_)| = 3.2 kJ/mol and |Δ*E*_int_(C_18_H_38_)| – 2 × |Δ*E*_int_(C_9_H_20_)| = 3.6 kJ/mol, respectively. This effect can be visualized by considering an *n*-butane dimer as two ethane-dimer-like units in direct contact (Scheme 1). Compared to the total interaction energy (blue dashed lines) of two isolated ethane dimers, this butane dimer not only retains all the individual van der Waals interactions present in the isolated units but also introduces new cross-interactions (red dashed lines) between these adjacent units. These new contact points cause the total interaction energy of the butane dimer to exceed the sum of the interaction energies of two isolated ethane dimers. Moreover, the synergistic cooperativity becomes more pronounced with increasing chain length, as a longer alkane chain provides more contact points for such cross-interactions. However, the effect eventually reaches an upper limit owing to the short-range nature of the van der Waals interaction, as distal segments become so far apart that they cannot contribute significantly.

**Scheme 1:**
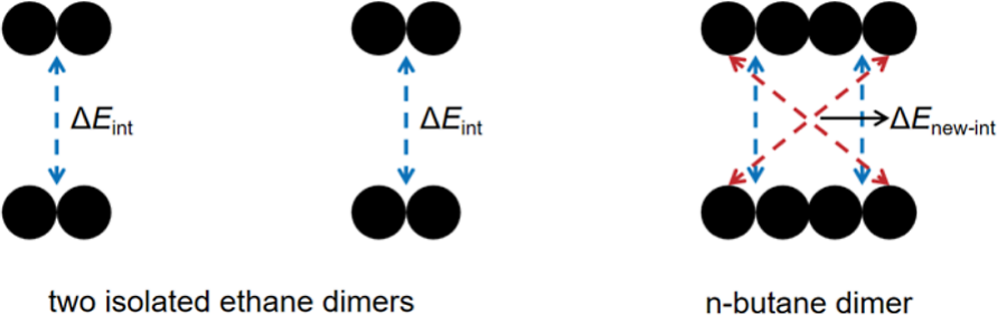
Synergistic Cooperativity in an *n*-Butane Dimer

The overall dependence of Δ*E*_int_ on the chain length (*n*) is linear, despite a subtle odd–even oscillation. A near-perfect linear fit (*R* ^2^ = 0.9995) gives the equation Δ*E*_int_ = −3.6*n* + 2.2 in this work (Figure 3). Comparison with the previous MP2/CBS ref 21 (Δ*E*_int_ = −3.8*n* + 2.7, *R* ^2^ = 0.9995) indicates that the model of this work provides a more accurate quantitative description. The improvement stems from the superior description of dispersion interactions afforded by the DLPNO–CCSD­(T1)/CBS method. The agreement of our slope with the average incremental gain not only confirms the constant and cumulative nature of the van der Waals interactions but also provides a more reliable quantitative metric. Consequently, this linear model serves as a robust reference for predicting interaction energies in larger *n*-alkane dimers.

**Figure 3:**
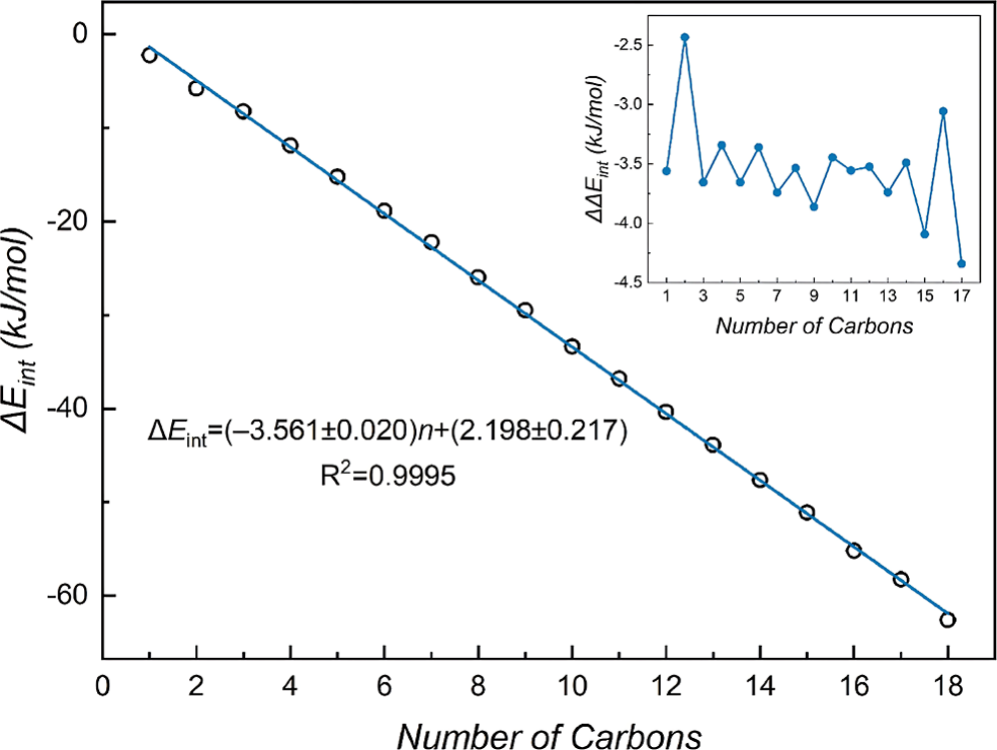
Linear correlation between the interaction energy (Δ*E*_int_, kJ/mol) and the number of carbons (*n*). The odd–even oscillation phenomenon is given as an inset on the graph.

### 3.5 Interaction Properties: LED Analysis

Having established the robust linear relationship between interaction energy and chain length, the physical origins of these interactions were further investigated. Since the LED method is not applicable at the DLPNO–CCSD­(T1)/CBS­(aug2/3)//M05-2X-D3/6-31G** level of theory employed here, an alternative strategy was adopted to decompose the interaction energy using the direct calculation of its key components at the same level. These components include the binding energy (Δ*E*_bind_), the geometric preparation energy (Δ*E*_geo‑prep_), the BSSE-uncorrected interaction energy (Δ*E*_raw‑int_), the BSSE correction (Δ*E*_BSSE_), as well as the Δ*E*_HF_ and Δ*E*_C_ contributions, all of which are presented in Table 3.

**Table 3:** Energy Decomposition of *n*-Alkane (C_*n*_H_2*n*+2_, *n* = 1–18) Dimer Interactions at the DLPNO–CCSD­(T1)/CBS­(aug2/3)//M05-2X-D3/6-31G** Level^a^

dimer	Δ*E*_bind_	Δ*E*_geo‑prep_	Δ*E*_raw‑int_	Δ*E*_BSSE_	Δ*E*_int_	Δ*E*_HF_	Δ*E*_C_	|Δ*E*_BSSE_/Δ*E*_int_|	Δ*E*_C_/Δ*E*_int_
*n* = 1	–2.2	0.0	–2.5	–0.3	–2.2	1.9	–4.1	11.7	185.1
*n* = 2	–5.8	0.0	–5.9	–0.1	–5.8	5.3	–11.1	1.2	192.3
*n* = 3	–8.2	0.0	–8.1	0.1	–8.2	6.2	–14.4	1.8	175.4
*n* = 4	–11.8	0.0	–11.7	0.2	–11.9	8.8	–20.7	1.5	174.3
*n* = 5	–15.2	0.0	–14.8	0.4	–15.2	11.0	–26.2	2.7	172.0
*n* = 6	–18.8	0.1	–18.4	0.5	–18.9	13.5	–32.3	2.6	171.3
*n* = 7	–22.2	0.1	–21.4	0.8	–22.2	15.6	–37.8	3.8	170.1
*n* = 8	–25.9	0.1	–25.1	0.9	–26.0	18.1	–44.0	3.3	169.5
*n* = 9	–29.5	0.0	–28.3	1.2	–29.5	21.0	–50.5	4.1	171.1
*n* = 10	–33.2	0.2	–32.4	1.0	–33.4	23.3	–56.6	3.1	169.7
*n* = 11	–36.8	0.1	–35.6	1.2	–36.8	25.2	–62.1	3.2	168.5
*n* = 12	–40.2	0.1	–38.8	1.6	–40.4	27.3	–67.7	3.9	167.7
*n* = 13	–43.8	0.1	–42.1	1.8	–43.9	29.6	–73.5	4.1	167.5
*n* = 14	–47.4	0.2	–45.7	1.9	–47.6	32.5	–80.1	4.1	168.2
*n* = 15	–51.1	0.0	–48.9	2.2	–51.1	34.4	–85.5	4.4	167.2
*n* = 16	–54.8	0.4	–52.5	2.7	–55.2	38.2	–93.4	4.8	169.2
*n* = 17	–58.3	0.0	–55.7	2.5	–58.3	38.8	–97.1	4.4	166.6
*n* = 18	–62.1	0.5	–59.8	2.9	–62.6	43.8	–106.4	4.6	170.0

a Units are kJ/mol unless otherwise noted. |Δ*E*_BSSE_/Δ*E*_int_| and Δ*E*_C_/Δ*E*_int_ are given in percentages (%).

The Δ*E*_geo‑prep_ is positive and negligible across all systems, with a maximum value of only 0.5 kJ/mol for the C_18_H_38_ dimer (Table 3). This indicates that the energy penalty for geometric deformation of the monomers upon dimer formation is subtle, implying that their structures remain largely unperturbed. Furthermore, as Δ*E*_bind_ is the sum of Δ*E*_int_ and the negligible Δ*E*_geo‑prep_, Δ*E*_bind_ serves as an excellent approximation for Δ*E*_int_.

The magnitude and sign of the Δ*E*_BSSE_, exhibit a dependence on chain length. For the CH_4_ and C_2_H_6_ dimers, ΔE_BSSE_ is negative, indicating that neglecting BSSE would lead to an overestimation of the attractive interaction (i.e., a more negative Δ*E*_int_). In contrast, for all larger dimers (*n* ≥ 3), Δ*E*_BSSE_ is positive, meaning that neglect of BSSE would result in an underestimation of the attractive interaction. Critically, while the absolute value of Δ*E*_BSSE_ increases with chain length, its relative importance is best assessed by the ratio |Δ*E*_BSSE_/Δ*E*_int_|, which represents the relative error introduced by neglecting this correction. This ratio remains below 4.8% for all dimers larger than methane, despite the increasing absolute error. The CH_4_ dimer is a slight outlier with a ratio of 11.7%, which is attributable to the challenges of describing weak interactions in very small systems with diffuse functions. This analysis demonstrates that for the large aug-cc-pVXZ (X = D, T) basis sets employed, the absolute BSSE is nonzero and grows with system size, but the relative error remains small. Consequently, for the qualitative trends discussed in this study, Δ*E*_raw‑int_ provides a reasonable approximation to the fully corrected Δ*E*_int_. However, for quantitative accuracy, especially in benchmark studies aiming at chemical accuracy (∼1 kJ/mol), explicitly including the BSSE correction to obtain Δ*E*_int_ is strongly recommended.

The Δ*E*_HF_ and Δ*E*_C_ components reveal their opposing roles. The Δ*E*_HF_ term is destabilizing (positive) for all dimers, while the Δ*E*_C_ term is the primary source of attraction (negative). The magnitude of both terms increases with chain length. The ratio Δ*E*_C_/Δ*E*_int_ ranges from 167% to 192%, indicating that the correlation energy must not only overcome the Pauli repulsion represented by Δ*E*_HF_ but also provides the net stabilizing interaction. This unambiguously confirms that correlation–driven interactions, predominantly dispersion,^27,63,64^ are the dominant force governing the binding in *n*-alkane dimers.

Moreover, the nondispersion components of the interaction are found to be negligible (approaching zero), confirming that the interaction can be treated as exclusively between nonpolar molecules. The dominant role of London dispersion forces is further corroborated by a detailed LED analysis^19^ (see Supporting Information).

### 3.6 Interaction Thermodynamics: Enthalpy & Entropy

Building upon the understanding of the interaction energies and their components, the relevant thermodynamic parameters, namely the enthalpy change (Δ*H*) and Gibbs free energy change (Δ*G*) for the dimerization process, were further computed at 298.15 and 100 K (Figure 4). The competition between steric repulsion and van der Waals attraction suggests a crossover in *n*-alkanes to a folded ground state beyond a certain length. Goodman^65^ first probed this crossover point, but the theoretical methods used at that time were limited in evaluating dispersion. Later, a study combining Raman spectroscopy with theoretical calculations suggested that C_17_H_36_ is the longest alkane that still prefers a linear conformation at low temperatures.^66^ Subsequently, the DLPNO–CCSD­(T) method combined with thermodynamic corrections confirmed that the last linear conformer at 100 K should be either C_16_H_34_ or C_17_H_36_ with C_17_H_36_ being most probable.^27^ In this connection, our approach reproduces this conclusion (see Computational Strategy of Thermodynamic Corrections in the Supporting Information). Therefore, we expect our results for the dimerization process to align with subsequent experimental observations.

**Figure 4:**
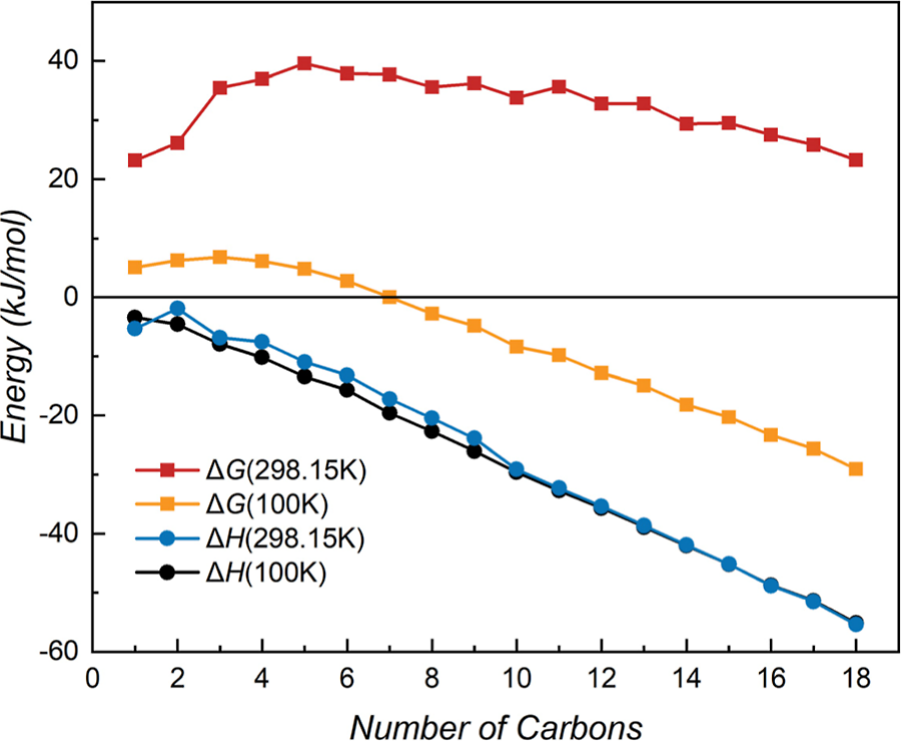
Δ*H* and Δ*G* (in kJ/mol) for dimer interactions of *n*-alkanes (C_*n*_H_2*n*+2_, *n* = 1–18).

At 100 and 298.15 K temperatures, the negative Δ*H* values confirm that the dimerization is exothermic (Figure 4). Overall, the absolute values of the Δ*H* increase with carbon chain length, indicating a greater release of heat for longer-chain alkanes. Notably, the exothermicity is slightly larger at 100 K than at 298.15 K for *n* = 2–15, which can be attributed to enhanced van der Waals interactions owing to reduced thermal motion at cryogenic temperatures. Conversely, an inverse trend is observed for *n* = 1 and *n* = 16–18, suggesting that these dimers may adopt distinct conformational arrangements at 100 K.

Analysis of the Gibbs free energy reveals the temperature dependence of spontaneity. At 298.15 K, all Δ*G* values are positive, indicating that the dimerization is not spontaneous across the entire series. For shorter chains (*n* = 1–5), Δ*G* becomes more positive with increasing *n*, while for longer chains (*n* = 6–18), Δ*G* overall decreases, which suggests that the tendency for spontaneous reaction increases with chain elongation. In contrast, a clear transition in spontaneity is observed at 100 K. The Δ*G* values are positive for shorter chains (*n* = 1–7) but become negative for longer chains (*n* = 8–18). The Δ*G* monotonically decreases with chain length for *n* = 4–18, shifting from positive to negative. These results indicate that the spontaneous dimerization starts from *n* ≥ 8 at 100 K.

## 4 Conclusions and Future Prospects

This work focused on the dimerization of the *n*-alkanes, C_*n*_H_2*n*+2_, *n* = 1–18. The geometry optimization methods suitable for this system were initially explored followed by analysis of the characteristics of the optimized structures. Subsequently, the calculation methods for interaction energy were investigated followed by calculation of the interaction energies of the *n*-alkane dimers to reveal the nature of the interactions. Finally, the thermodynamic properties of the interactions of the *n*-alkane dimers were studied.

The M05-2X-D3/6-31G** method demonstrates good accuracy and cost-effectiveness in the geometry optimization of *n*-alkane dimers, making it a promising new approach worthy of consideration. The equilibrium distances of CH_4_ and C_2_H_6_ dimers are relatively small (3.658 Å and 3.688 Å, respectively), while the equilibrium distances for larger dimers (*n* ≥ 3) lie within a range of 3.899–4.056 Å. A clear odd–even difference is observed among these larger dimers.

The DLPNO–CCSD­(T1)/CBS­(aug2/3) method exhibits extremely high accuracy in calculating the interaction energies of *n*-alkane dimers. This method was employed to calculate the interaction energies of *n*-alkane (C_*n*_H_2*n*+2_, *n* = 1–18) dimers, and the BSSE-corrected results are reported from −2.2 kJ/mol (*n* = 1) to −62.6 kJ/mol (*n* = 18). In addition, for quantitative accuracy, especially in benchmark studies aiming at chemical accuracy (∼1 kJ/mol), explicitly including the BSSE correction to obtain interaction energies is strongly recommended. The overall dependence of the interaction energy (Δ*E*) on the chain length (*n*) is linear, as quantified by the equation Δ*E*(*n*) = −3.6*n* + 2.2 kJ/mol, despite a subtle odd–even oscillation.

The energy penalty for geometric deformation of the monomers upon dimer formation is tiny, implying that their structures remain largely unperturbed. Correlation-driven interactions, predominantly dispersion, are the dominant force governing the binding in *n*-alkane dimers. Moreover, the nondispersion components of the interaction are found to be negligible (approaching zero), confirming that the interaction can be treated as exclusively between nonpolar molecules. Both carbon chain length and temperature are critical factors controlling the spontaneity of *n*-alkane dimerization. Thermodynamic results indicate that spontaneous dimerization starts from *n* ≥ 8 at 100 K.

This work has achieved precise calculation of the interaction energies of *n*-alkane dimers by investigating geometry optimization methods and single-point energy calculation approaches, thereby unveiling the essence of their intermolecular interactions and associated thermodynamic properties. More importantly, our findings not only show that the role of van der Waals interactions in molecular chemistry has been severely underestimated,^2,3^ but also highlight the immense potential of London dispersion as a predictable tool for the rational design of novel molecules and chemical reactions. A mechanistic study on the Corey-Bakshi-Shibata reduction confirms this, with a 24.7 kJ/mol dispersion energy difference between diastereomeric transition states dominating the enantioselectivity.^5^

## Supplementary Material





## Notes

Alkane dimers ZZ&LP 3-26 clean (PDF)
Detailed computational strategy of thermodynamic corrections, benchmark results for the methane dimer, NCI plots of larger *n*-alkane (C_*n*_H_2*n*+2_, *n* = 7–18) dimers, computational cost comparison: DLPNO–CCSD­(T1)/CBS vs DLPNO–CCSD­(T)/CBS, and results from the LED analysis at DLPNO–CCSD­(T)/aug-cc-pVTZ//M05-2*X*/6-31G** level (DOC). Separate file of the Cartesian coordinates for *n*-alkane (C_*n*_H_2*n*+2_, *n* = 1–18) dimer structures used in this article (XYZ)
