# Supplementary material for: Intermolecular Interactions of Linear Alkane (N ≤ 18) Dimers: A High Accuracy Theoretical Study
Source: ACS Omega. 2026 May 4;11(19):27875–83. doi: 10.1021/acsomega.5c11053 (PMC13191556; doi:10.1021/acsomega.5c11053)
Supplement: Supplementary file 1 [file ao5c11053_si_001.pdf]

Supporting Information

Intermolecular Interactions of Linear Alkane ($n \leq$ 18) Dimers: A High Accuracy Theoretical Study

Chenhui Wang,[a] WanYing Huang,[a] Liang Pu,[a] Zhong Zhang,[a] Robert Bruce King*[b]*

[a]College of Chemistry & Pharmacy, Northwest A&F University, Yangling, Shaanxi 712100, P. R. China

[b]Department of Chemistry and Center for Computational Chemistry, University of Georgia, Athens, Georgia 30602, USA

e-mails: zhangzhong6@126.com; rbking@uga.edu

Computational Strategy of Thermodynamic Corrections

This work has demonstrated that the DLPNO-CCSD(T1)/CBS(aug2/3)//M05-2X-D3/6-31G** method is both computationally accurate and robust for studying *n*-alkane dimers. For thermodynamic corrections, using the same M05-2X-D3/6-31G** method would be a convenient choice. However, its accuracy for this purpose requires careful evaluation.

When Liakos and Neese [1] theoretically explored the question of which straight-chain *n*-alkane has the longest carbon chain at 100 K, they calculated the Gibbs free energy change ($G_{\text{hairpin}} - G_{\text{extended}}$) for the transformation of *n*-alkanes from a straight-chain structure to a hairpin structure at 100 K. The main results are as follows: the value of $G_{\text{hairpin}} - G_{\text{extended}}$ is approximately 0.9 kJ/mol for $C_{16}H_{34}$, –0.4 kJ/mol for $C_{17}H_{36}$, and –3.7 kJ/mol for $C_{18}H_{38}$. When calculating thermodynamic corrections, they employed the correction factor proposed by Truhlar et al. [2] to correct the zero-point energy and calculated the contribution of vibrational entropy according to Grimme's scheme [3] (using the recommended ω_0 = 100^{-1} cm and α = 4.0). This work adopted their strategies for calculating zero-point energy and vibrational entropy when computing thermodynamic corrections with the M05-2X-D3/6-31G** method. Their reported thermodynamic results are then used as a reference to evaluate the accuracy of thermodynamic correction schemes when combined with the DLPNO-CCSD(T1)/CBS electronic energies employed in this work.

When calculating thermodynamic corrections using the M05-2X-D3/6-31G** [4-8] method, this work also adopted the correction factor proposed by Truhlar et al. and followed Grimme's scheme. Since there is no dedicated correction factor for the 6-31G** basis set, we thus used the correction factor (0.961) proposed for the 6-31+G** basis set. Given that the correction factors of DFT methods usually do not change significantly with the basis set, we believe that the

thermodynamic results obtained using this correction factor will be closer to the experimental values than the uncorrected ones. The thermodynamic results are shown in Figure S1a.

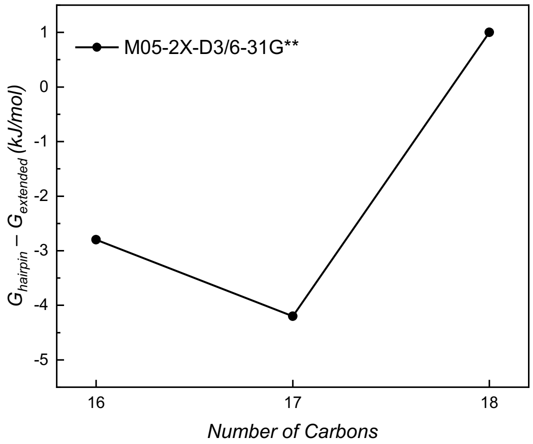

(a) $G_{\text{hairpin}} - G_{\text{extended}}$ obtained from thermodynamic correction calculations using the M05-2X-D3/6-31G** method.

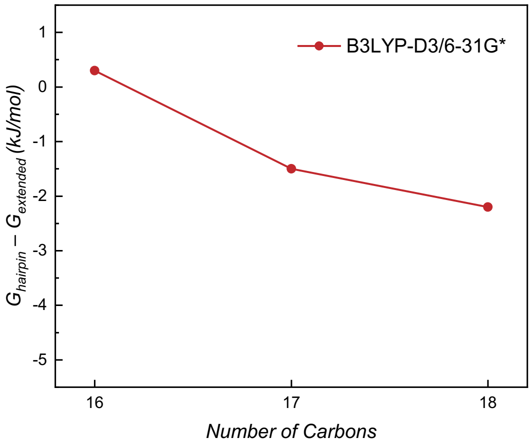

(b) $G_{\text{hairpin}} - G_{\text{extended}}$ obtained from thermodynamic correction calculations using the B3LYP-D3/6-31G* method.

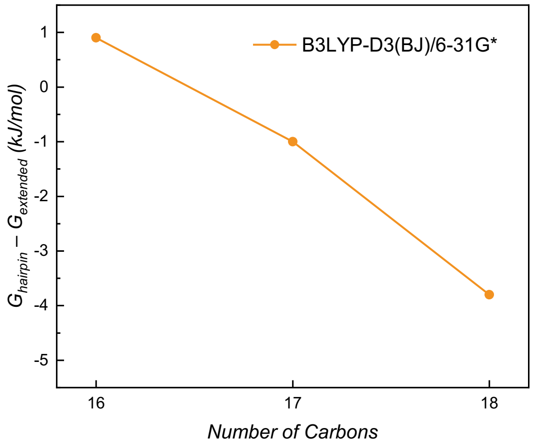

(c) $G_{\text{hairpin}} - G_{\text{extended}}$ obtained from thermodynamic correction calculations using the B3LYP-D3(BJ)/6-31G* method.

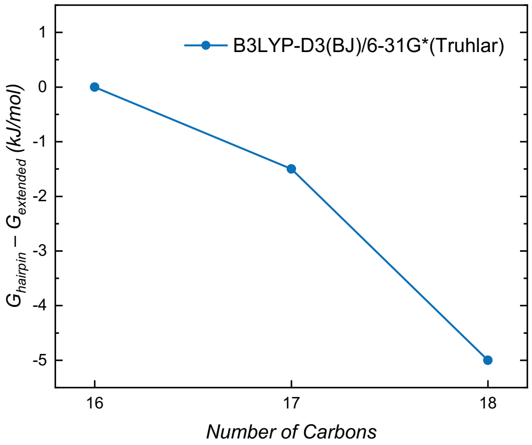

(d) $G_{\text{hairpin}} - G_{\text{extended}}$ obtained from thermodynamic correction calculations using the B3LYP-D3(BJ)/6-31G* method and Truhlar's scheme.

Figure S1. $G_{\text{hairpin}} - G_{\text{extended}}$ obtained from thermodynamic correction calculations using different strategies.

The $G_{\text{hairpin}} - G_{\text{extended}}$ value is –2.8 kJ/mol for $C_{16}H_{34}$, –4.2 kJ/mol for $C_{17}H_{36}$, and 1.0 kJ/mol for $C_{18}H_{38}$ (Figure S1a). These results show significant differences compared to the

Neese's results, with absolute errors of 3.7, 3.8, and 4.7 kJ/mol, respectively. Moreover, the trend is not reproduced: $C_{18}H_{38}$ is predicted to favor the extended conformation (positive value), contrary to the Neese's results. This indicates that using the M05-2X-D3/6-31G** method for thermodynamic corrections may not yield accurate results for this specific conformational equilibrium, highlighting the need to explore alternative approaches.

This work first considered the PW6B95-D3/def2-QZVP method used by Liakos and Neese. However, the extremely high computational cost of this method renders it prohibitive for larger systems. Subsequently, out of economic considerations, the B3LYP-D3/6-31G* [9,10] method was employed. The correction factor (0.977) proposed by Truhlar et al. was adopted, and Grimme's scheme was also followed. The thermodynamic results are shown in Figure S1b.

The $G_{\text{hairpin}} - G_{\text{extended}}$ value is 0.3 kJ/mol for $C_{16}H_{34}$, –1.5 kJ/mol for $C_{17}H_{36}$, and –2.2 kJ/mol for $C_{18}H_{38}$ (Figure S1b). Compared to the Neese's results, the absolute deviations are 0.6, 1.1, and 1.5 kJ/mol, respectively. These deviations are within the 0.5–2.0 kJ/mol combined uncertainty of the electronic energy (~0.5 kJ/mol) and thermodynamic corrections (1–1.5 kJ/mol) estimated by Neese [1]. Moreover, the trend is correctly captured: $C_{16}H_{34}$ near zero, $C_{17}H_{36}$ negative, and $C_{18}H_{38}$ more negative. This performance sparks our interest in further exploring the potential of this method.

The study by Grimme et al. [11] indicates that the Becke-Johnson damping has more significant advantages in estimating intramolecular dispersion interactions. Therefore, this work switched to using the B3LYP-D3(BJ)/6-31G* method to calculate thermodynamic corrections. The correction factor proposed by Truhlar et al. was also adopted, and Grimme's scheme was also followed. The thermodynamic results are shown in Figure S1c.

The $G_{\text{hairpin}} - G_{\text{extended}}$ value is 0.9 kJ/mol for $C_{16}H_{34}$, –1.0 kJ/mol for $C_{17}H_{36}$, and –3.8 kJ/mol for $C_{18}H_{38}$ (Figure S1c). Compared to the Neese's results, the absolute deviations are 0.0, 0.6, and 0.1 kJ/mol, respectively. These deviations are within the 0.5–2.0 kJ/mol uncertainty range estimated by Neese [1]. Notably, the result for $C_{16}H_{34}$ exactly matches the Neese's result, and the values for $C_{17}H_{36}$ and $C_{18}H_{38}$ are also in excellent agreement. Although the overall trend is captured, the slope deviates from the Neese's results: the drop from $C_{16}H_{34}$ to $C_{17}H_{36}$ is overestimated (–1.9 vs. –1.3 kJ/mol), while the drop from $C_{17}H_{36}$ to $C_{18}H_{38}$ is underestimated (–2.8 vs. –3.3 kJ/mol). This suggests that further improvement is required to capture the precise trend across the series.

This work initially adopted Grimme's scheme to handle the contribution of vibrational entropy. As the research progressed, we found that replacing Grimme's scheme with the one proposed by Truhlar et al. [12] (with a frequency threshold set at 100 cm^{-1}) could significantly improve the consistency with the reference trend, as illustrated in Figure S1d.

The $G_{\text{hairpin}} - G_{\text{extended}}$ value is 0.0 kJ/mol for $C_{16}H_{34}$, –1.5 kJ/mol for $C_{17}H_{36}$, and –5.0 kJ/mol for $C_{18}H_{38}$ (Figure S1d). Compared to the Neese's results, the absolute deviations are 0.9, 1.1, and 1.3 kJ/mol, respectively. These deviations are within the 0.5–2.0 kJ/mol uncertainty range estimated by Neese [1]. Moreover, the drops from $C_{16}H_{34}$ to $C_{17}H_{36}$ (–1.5 vs. –1.3 kJ/mol) and from $C_{17}H_{36}$ to $C_{18}H_{38}$ (–3.5 vs. –3.3 kJ/mol) are both slightly overestimated, but their slope is much closer to the Neese's results than in Grimme's scheme. This indicates that the Truhlar scheme better captures the gradual transition across the alkane series. Given that the primary objective is to reliably identify the crossover point from linear to folded conformers, rather than to minimize numerical deviations for individual points, the Truhlar scheme is considered the most suitable choice for calculating thermodynamic corrections in this work.

Benchmark Results for the Methane Dimer

A comprehensive set of quantum chemical method and basis set combinations was employed to optimize the methane dimer geometry and evaluate their performance. The tested theoretical methods included the following: the density functionals B3LYP-D3, M05-D3 [13], M05-2X-D3, M06-L-D3 [14], M06-2X-D3 [15], B2PLYP-D3 [16]; and the wave function-based method MP2 [17,18]. The following basis sets were used with the aforementioned methods: 6-31G**, 6-311++G** [19,20], DZVP [21,22], cc-pVDZ [23], cc-pVTZ [23], aug-cc-pVTZ [23,24]. In this benchmarking study, the CCSD(T) [25] method in combination with the aug-cc-pVTZ basis set was used to compute the reference value for the equilibrium distance (R_e). The deviations (Δ) of the predicted R_e values for all method/basis set combinations from the CCSD(T)/aug-cc-pVTZ reference value are reported in Table S1.

Table S1. Deviations (Δ) of Methane Dimer Equilibrium Distances (*R*e) from the CCSD(T)/aug-cc-pVTZ Reference Value[a].

	6-31G**	6-311++G**	DZVP	cc-pVDZ	cc-pVTZ	aug-cc-pVTZ
B3LYP-D3	–0.132	–0.032	–0.029	–0.132	–0.034	–0.034
M05-D3	–0.134	–0.121	–0.051	–0.117	–0.037	–0.121
M05-2X-D3	–0.031	–0.034	0.014	–0.025	–0.036	–0.036
M06-L-D3	0.091	0.068	0.078	0.091	0.074	0.067
M06-2X-D3	–0.170	–0.159	–0.031	–0.130	–0.130	–0.069
B2PLYP-D3	–0.034	–0.032	–0.029	–0.025	–0.036	–0.036
MP2	0.134	0.117	0.185	0.098	0.028	–0.037

[a] The reference R_e value computed at the CCSD(T)/aug-cc-pVTZ level is 3.689 Å.

The Δ values in Table S1 reveal a significant spread in predicted R_e values. Specifically, the MP2/DZVP method shows the largest positive Δ (0.185 Å), while the M06-2X-D3/6-31G** method shows the largest negative Δ (–0.170 Å). This indicates that the predicted R_e value is

highly sensitive to the choice of theoretical method and basis set. Since methane represents the simplest *n*-alkane, this finding is likely applicable to more complex *n*-alkane dimers.

Different theoretical methods exhibit dramatically different dependencies on the basis set, as quantified by the range (max – min) of their Δ values. Specifically, the ranges are: 0.103 Å (B3LYP-D3), 0.097 Å (M05-D3), 0.050 Å (M05-2X-D3), 0.024 Å (M06-L-D3), 0.139 Å (M06-2X-D3), 0.011 Å (B2PLYP-D3), and 0.222 Å (MP2). This demonstrates that the predicted structures from some methods (e.g., M06-2X-D3 and MP2) are far more sensitive to the basis set than others (e.g., B2PLYP-D3 and M06-L-D3).

Although the M05-2X-D3/DZVP combination yields the smallest absolute Δ (0.014 Å), this study selected the M05-2X-D3/6-31G** method (Δ = –0.031 Å) for all subsequent studies. This decision is based on two key considerations: First, the M05-2X-D3 functional itself exhibits low sensitivity to the basis set, meaning both combinations offer high accuracy comparable to the CCSD(T)/aug-cc-pVTZ reference and maintain low computational cost. Second, the 6-31G** basis set is a more robust choice than the DZVP basis set; it incorporates polarization functions that provide a superior description of molecular electronic structure and properties, making it better suited for extending this study to larger *n*-alkane systems.

NCI Plots of Larger *n*-Alkane (C_nH_{2n+2}, *n* = 7–18) Dimers

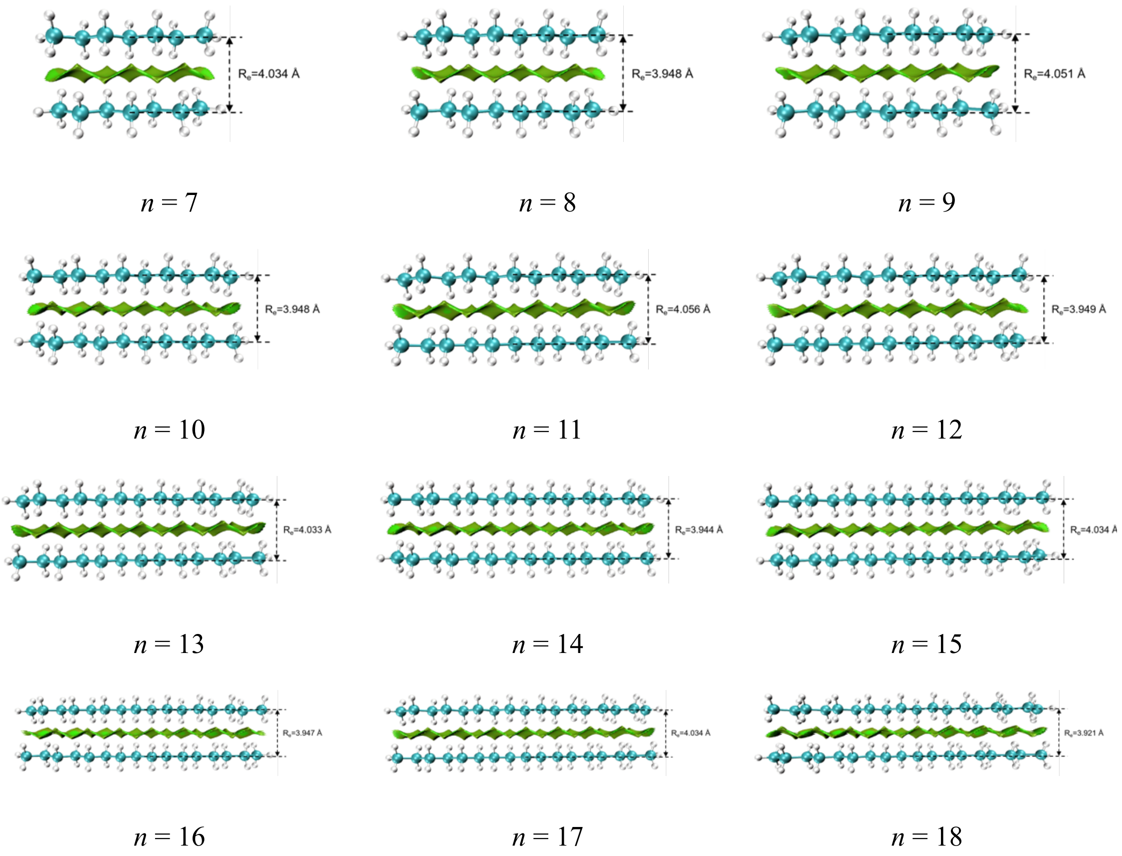

Figure S2. NCI plots of larger *n*-alkane (C_nH_{2n+2}, n = 7-18) dimers. The equilibrium distances were given in angstrom (Å). The color scale is based on sign(λ_2)ρ, ranging from blue (strong attraction) through green (weak dispersion) to red (strong repulsion).

Computational Cost Comparison: DLPNO-CCSD(T1)/CBS vs. DLPNO-CCSD(T)/CBS

Table S2. Computational cost (in min) comparison of *n*-alkane (C_nH_{2n+2}, n = 1–4) dimers with all other conditions fixed: DLPNO-CCSD(T1)/CBS vs. DLPNO-CCSD(T)/CBS.

Method	n = 1	n = 2	n = 3	n = 4
DLPNO-CCSD(T1)/CBS[a]	5	34	131	189
DLPNO-CCSD(T)/CBS[a]	2	15	60	136

[a] Basis set is aug-cc-pVXZ (X = D, T).

LED Analysis at DLPNO-CCSD(T)/aug-cc-pvTZ//M052X/6-31G** Level

To further elucidate the precise nature of the correlation interaction, a LED analysis was performed at the DLPNO-CCSD(T)/aug-cc-pVTZ//M052X/6-31G** level.

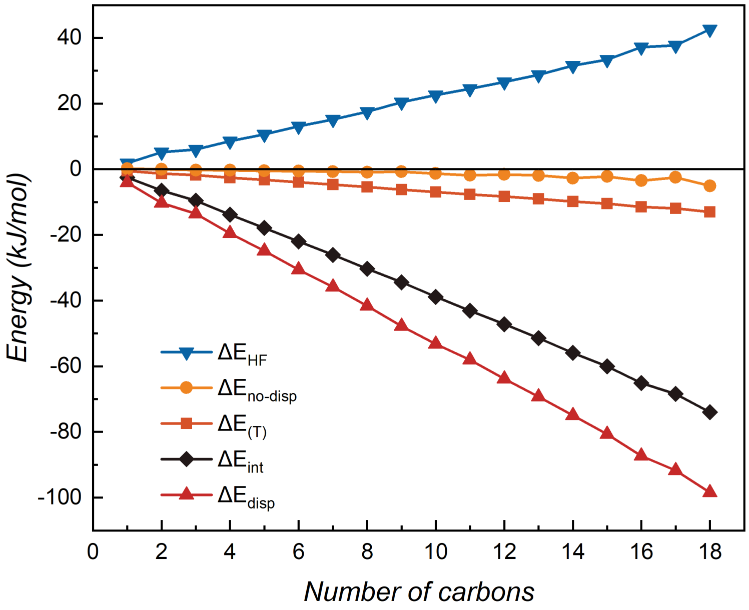

Figure S3. LED decomposition of the interaction energy (ΔE_{int}) for *n*-alkane (C_nH_{2n+2}, n = 1–18) dimers.

The results robustly corroborate and extend the conclusions from the main text. The repulsive ΔE_{HF} term is again overwhelmed by the attractive ΔE_{C}, yielding a net negative ΔE_{int} (Figure S2). More significantly, the LED decomposition reveals that the correlation energy is overwhelmingly dominated by the dispersion component (ΔE_{disp}). The non-dispersion contributions ($\Delta E_{\mathrm{non\text{-}disp}}$), which encompass electrostatic and induction forces, are negligible across the entire series, with values consistently approaching zero. This critical finding confirms that the interaction in *n*-alkane dimers is essentially a pure dispersion interaction between non-polar molecules. Given that $|\Delta E_{\mathrm{disp}}| >> |\Delta E_{\mathrm{non\text{-}disp}}|$ and $|\Delta E_{(\mathrm{T})}|$, it is conclusive that London dispersion forces are the definitive physical origin of the attraction in *n*-alkane dimers. Furthermore, the interaction energies obtained from this method replicate the synergistic

cooperativity [26] observed in our primary DLPNO-CCSD(T)/CBS(aug2/3)//M052X/6-31G** calculations, with the binding of even *n*-alkane dimers being enhanced relative to twice that of their half-length counterparts (Table S2). The reproducibility of this effect across independent high-level computational methods confirms it as a robust physical phenomenon, not an artifact of a single method.

Table S3. Interaction energies (ΔE_{int}) of *n*-alkane (C_nH_{2n+2}, n = 1-18) dimers calculated at DLPNO-CCSD(T)/aug-cc-pVTZ//M052X/6-31G** level.

Dimer	n = 1	n = 2	n = 3	n = 4	n = 5	n = 6	n = 7	n = 8	n = 9
ΔE_{int}	–2.5	–6.5	–9.6	–13.8	–17.9	–22.0	–26.1	–30.3	–34.4
Dimer	n = 10	n = 11	n = 12	n = 13	n = 14	n = 15	n = 16	n = 17	n = 18
ΔE_{int}	–38.8	–43.0	–47.2	–51.4	–56.0	–60.0	–65.1	–68.4	–74.0

Literature References

[1] Liakos D G, Neese F. Domain based pair natural orbital coupled cluster studies on linear and folded alkane chains[J]. *J. Chem. Theory Comput.* **2015**, 11, 2137-2143.

[2] Alecu I M, Zheng J J, Zhao Y, Truhlar D G. Computational thermochemistry: scale factor databases and scale factors for vibrational frequencies obtained from electronic model chemistries[J]. *J. Chem. Theory Comput.* **2010**, 6, 2872-2887.

[3] Grimme S. Supramolecular Binding Thermodynamics by Dispersion-Corrected Density Functional Theory[J]. *Chem-Eur. J.* **2012**, 18, 9955-9964.

[4] Zhao Y, Schultz N E, Truhlar D G. Design of Density Functionals by Combining the Method of Constraint Satisfaction with Parametrization for Thermochemistry, Thermochemical Kinetics, and Noncovalent Interactions[J]. *J. Chem. Theory Comput.* **2006**, 2, 364.

[5] Grimme S, Antony J, Ehrlich S, Krieg H. A consistent and accurate ab initio parametrization of density functional dispersion correction (DFT-D) for the 94 elements H-Pu[J]. *J. Chem. Phys.* **2010**, 132, 154104.

[6] Ditchfield R, Hehre W J, Pople J A. Self-Consistent Molecular-Orbital methods. IX. an extended Gaussian-Type basis for Molecular-Orbital studies of organic molecules[J]. *J. Chem. Phys.* **1971**, 54, 724-728.

[7] Hehre W J, Ditchfield R, Pople J A. Self-Consistent Molecular Orbital Methods. XII. Further Extensions of Gaussian-Type Basis Sets for Use in Molecular Orbital Studies of Organic Molecules[J]. *J. Chem. Phys.* **1972**, 56, 2257-2261.

[8] Hariharan P C, Pople J A. The influence of polarization functions on molecular orbital hydrogenation energies[J]. *Theor. Chim. Acta.* **1973**, 28, 213-222.

[9] Becke A D. Density-functional thermochemistry. III. The role of exact exchange[J]. *J. Chem. Phys.* **1993**, 98, 5648-5652.

[10] Stephens P J, Devlin F J, Chabalowski C F, Frisch M J. Ab Initio Calculation of Vibrational Absorption and Circular Dichroism Spectra Using Density Functional Force Fields. *J. Phys. Chem.* **1994**, 98, 11623-11627.

[11] Grimme S, Ehrlich S, Goerigk L. Effect of the damping function in dispersion corrected density functional theory[J]. *J. Comput. Chem.* **2011**, 32, 1456-1465.

[12] Ribeiro R F, Marenich A V, Cramer C J, Truhlar D G. Use of solution-phase vibrational frequencies in continuum models for the free energy of solvation[J]. *J. Phys. Chem. B* **2011**, 115, 14556-14562.

[13] Zhao Y, Schultz N E, Truhlar D G. Exchange-correlation functional with broad accuracy for metallic and nonmetallic compounds, kinetics, and noncovalent interactions[J]. *J. Chem. Phys.* **2005**, 123, 161103.

[14] Zhao Y, Truhlar D G. A new local density functional for main-group thermochemistry, transition metal bonding, thermochemical kinetics, and noncovalent interactions[J]. *J. Chem. Phys.* **2006**, 125, 194101.

[15] Zhao Y, Truhlar D G. The M06 suite of density functionals for main group thermochemistry, thermochemical kinetics, noncovalent interactions, excited states, and transition elements: two new functionals and systematic testing of four M06-class functionals and 12 other functionals[J]. *Theor. Chem. Acc.*, **2008**, 120, 215-241.

[16] Grimme S. Semiempirical hybrid density functional with perturbative second-order correlation[J]. *J. Chem. Phys.* **2006**, 124, 034108-034116.

[17] Møller C, Plesset M S. Note on an approximation treatment for Many-Electron systems[J]. *Phys. Rev.* **1934**, 46, 618-622.

[18] Binkley J S, Pople J A. Mller–Plesset theory for atomic ground state energies[J]. *Int. J. Quantum Chem.* **1975**, 9, 229-236.

[19] Krishnan R, Binkley J S, Seeger R, Pople J A. Self-consistent molecular orbital methods. XX. A basis set forcorrelated wave functions[J]. *J. Chem. Phys.* **1980**, 72, 650-654.

[20] Clark T, Chandrasekhar J, Spitznagel G W, Paul V R. Efficient diffuse function-augmented basis sets for anion calculations. III. The 3-21+G basis set for first-row elements, Li-F[J]. *J. Comput. Chem.* **1983**, 4, 294-301.

[21] Feller, D. The role of databases in support of computational chemistry calculations[J]. *J. Comput. Chem.* **1996**, 13, 1571-1586.

[22] Schuchardt K L, Didier B T, Elsethagen T, Sun L S, Gurumoorthi V, Chase J, Li J, Windus T L. Basis set exchange: A community database for computational sciences[J]. *J. Chem. Inf. Model.* **2007**, 47, 1045-1052.

[23] Dunning T H. Gaussian basis functions for use in correlated molecular calculations. I. The atoms B through Ne and H[J]. *J. Chem. Phys.* **1989**, 90, 1007-1023.

[24] Kendall R A, Dunning T H, Harrison R J. Electron affinities of the first-row atoms revisited. Systematic basis sets and wave functions[J]. *J. Chem. Phys.* **1992**, 96, 6796-6806.

[25] Raghavachari K, Trucks G W, Pople J A, Gordon M H. A Fifth-Order perturbation comparison of electron correlation theories[J]. *Chem. Phys. Lett.* **1989**, 157, 479-483.

[26] Mahadevi A S, Sastry G N. Cooperativity in noncovalent interactions[J]. *Chem. Rev.* **2016**, 116, 2775-2825.
